# Study on the Consumption of Non-Steroidal Anti-Inflammatory Drugs and Antibiotics by the Brazilian Adult Population: A Cohort Study

**DOI:** 10.3390/pharmacy12050150

**Published:** 2024-09-29

**Authors:** Douglas Araujo Pedrolongo, Fernanda Teixeira Sagioneti, Giovana Maria Weckwerth, Gabriela Moraes Oliveira, Carlos Ferreira Santos, Adriana Maria Calvo

**Affiliations:** 1 Department of Biological Sciences, Bauru School of Dentistry, University of São Paulo, Bauru 17012-901, Brazil; douglaspedrolongo@usp.br (D.A.P.); giovana.weckwerth@alumni.usp.br (G.M.W.); gab.moraes@usp.br (G.M.O.); cfsantos@fob.usp.br (C.F.S.); 2 Hospital for Rehabilitation of Craniofacial Anomalies, University of São Paulo, Bauru 17012-900, Brazil

**Keywords:** anti-bacterial agents, anti-inflammatory agents, non-steroidal, drug prescriptions, prescription drug misuse

## Abstract

Self-medication without a medical or dental prescription is an action that leads to a significant problems associated with the overuse of medication in Brazil. The inappropriate use of antibiotics and non-steroidal anti-inflammatory drugs (NSAIDs) leads to problems related to microbial agent resistance and gastrointestinal complications. The purpose of this study was to elucidate the patterns of antibiotic and NSAIDs consumption among the adult population of Brazil. The questionnaire was answered by 400 people residing in Brazil who had access to the link in the year 2023. The findings showed that approximately 89.5% of the volunteers had used NSAIDs, and 32.2% had used antibiotics whether or not these medications had been prescribed by doctors or dentists. It was noted that a large proportion of the adverse effects reported by the volunteers involved symptoms related to gastrointestinal complaints. There was a high prevalence of NSAIDs consumption in the studied population, which is consistent with the high frequency of risk of adverse reactions caused by these drugs, particularly in the gastrointestinal tract. In relation to antibiotics, it was observed that the non-prescription consumption of these medications by the population was considered high, reaching one-third of the total number of volunteers who consumed such medications.

## 1. Introduction

In the current era, there is a prevalent issue of overmedication, often attributed to self-medication. Self-medication is the act of using medication without a valid medical prescription or with outdated prescriptions. It involves individuals taking personal responsibility for their medication based on prevailing knowledge and beliefs without appropriate scientific oversight [1]. This practice is notably widespread in Brazil [2]. This practice has contributed to the emergence of numerous adverse effects, particularly the development of antibiotic-resistant bacterial strains.

Antimicrobial resistance stands as a prominent topic of contemporary discourse. Research has revealed a concerning upward trajectory in broad bacterial resistance, including among antibiotics categorized as “reserve antibiotics”, further underscoring its direct implications for quality of life, hospitalization frequencies, and mortality rates [3]. Antibiotics are pharmaceutical agents capable of inhibiting or eradicating the proliferation of bacterial infections by administration through the most diverse routes (i.e., oral, topical, or injections). In addition, they can be considered a non-renewable resource for the body since their effect is reduced with successive use [4]. The inappropriate use of antibiotics intensifies bacterial contact with the drugs, fostering increased mutation rates and genetic exchanges. This results in the integration of pre-existing resistant genes into novel microbial hosts, thereby augmenting the selective pressure process [5].

Non-steroidal anti-inflammatory drugs (NSAIDs) are widespread in their prescription and use, implemented across diverse healthcare settings for their analgesic and anti-inflammatory properties in the treatment of pain [6]. Nevertheless, despite their positive effects, this class of drugs can pose significant risks to the patient, due to possible side effects and interactions, especially when it comes to polypharmaceutical patients [7].

The risk of upper gastrointestinal (GI) complications, especially associated with the use of NSAIDs, is a serious public health problem around the world [8]. The risk profiles of traditional NSAIDs available over the counter vary considerably. Of particular concern are the combinations of two or more NSAIDs present in seemingly benign medications used for treating colds and flu [9,10]. Gathering population data on medication consumption, including the frequency of self-medication, is essential for evaluating the risk–benefit profile of NSAIDs. This information is crucial for making informed decisions regarding health policies [8,11].

The frequent use of NSAIDs has been shown to be associated with a two- to three-fold increase in gastrointestinal tract complications [11,12], risks of cardiovascular events [12,13], and risks of moderate to severe kidney injuries [14,15]. On the same day, a patient can take a cold medicine (i.e., combinations of paracetamol + antipyretics and nasal decongestants), an aspirin for a headache, and other combinations such as metamizole + muscle relaxants, potentially exceeding the recommended daily doses for NSAIDs, without being aware of doing so. This common practice in everyday life can significantly increase the risk of experiencing side effects from these medications.

In Brazil, there are currently over 50 different NSAIDs available in the pharmaceutical market. That said, the National Health Surveillance Agency (ANVISA), the authority responsible for protecting the health of the Brazilian population, has instituted regulations relating to the purchase of non-prescription medicines, including NSAIDs such as ibuprofen, naproxen, and diclofenac, which are sold over the counter. Nevertheless, some require a prescription from a health professional in order to be purchased, such as coxibs, for example [16,17]. Over-the-counter medications, which are available to the general public without the necessity of a formal medical or dental prescription, contribute to indiscriminate use due to unrestrained purchasing. It should be noted that the accessibility of these medications does not imply their exemption from adverse effects [18]. This contributes to a significant public health issue [8]. 

In Brazil, the Ministry of Health is responsible for implementing and regulating actions to promote and improve healthcare conditions, guaranteeing the efficacy and quality of medicines, as well as promoting rational use and access to medicines for the population [19]. The rational use of medicines was defined by the World Health Organization (WHO) in 1985 as the situation in which patients receive medicines appropriate to their clinical needs, in doses that meet their individual requirements, for an adequate period of time and at the lowest cost to them and their community [20]. However, some patients break these protocols by underusing or overusing these medications without realizing how harmful they can be.

WHO defines adherence as the degree to which people commit to recommendations agreed by health professionals, including taking medication. This definition is part of a treatment plan that corresponds to the implementation, prescription regimen, and interruption of pharmacotherapy [21]. This being said, the misuse of medications can lead to low patient adherence, which in turn poses a threat not only to human health but also to the health system as a whole [22].

As aforementioned, the irrational use of medications, including self-medication at this time, is prevalent in everyday social life, which indicates a worsening of human behavior in relation to reliable information from the healthcare system [23]. Given this context, it is essential to explore data regarding the most widely used medications among the general population, medications commonly prescribed by healthcare professionals, prescription methods, patient–clinician communication, common patient errors during treatment (such as incorrect timing of medication intake), and the prevalence of self-medication. This understanding is crucial for comprehending the clinical dynamics of medication use, including NSAIDs and antibiotics [2].

Brazil is ranked as the fifth country with the highest rate of self-medication. This raises concerns about the fragility of the public health system in the country, including a lack of personalized care, the mistreatment of patients, poor service quality, delayed and rushed care, and an overall impersonal approach to healthcare [24]. Furthermore, the extensive advertising of medications in easily accessible locations, coupled with the association with instant cures, and the shortcomings of the healthcare system are considered as potential contributing factors [25]. 

The objective of this study is to elucidate the patterns of antibiotic and NSAIDs consumption among the adult population of Brazil. This research will focus on a 90-day period preceding the survey and will encompass both prescribed usage and instances of self-medication within this demographic. Considering this, we are able to contemplate the proposal of health policies that aim to minimize instances of self-medication and the irrational utilization of medications [10].

## 2. Materials and Methods

The research was carried out by means of a survey on the consumption of antibiotics and NSAIDs by the adult Brazilian population during the period from June 2022 to June 2023, considering the 90 days prior to the survey. The survey defined which medicines were most used, which were prescribed by doctors or dentists, and which were consumed as self-medication, as well as the period in which they were taken and the frequency of consumption. This project was submitted to the Human Research Ethics Committee of the Bauru School of Dentistry, University of São Paulo (CEP/FOB/USP), and only began after its approval (CAAE#57817022.0.0000.5417).

### 2.1. Target Population

The survey was administered through the Google Forms platform and was completed by 400 individuals aged 18 and above residing in Brazil, who were invited to participate in the study.

Prior to the questionnaire, the volunteers who consented to take part in the study digitally signed the Free and Informed Consent Form, containing the form itself with information about the study, as well as subsequent fields where there was a question with two possible answers: ‘I have read and agree to take part in the study’ or ‘I do not agree to take part in the study’, followed by text boxes to insert each volunteer’s full name, identification document number, and email address, thus giving them permission to proceed with filling in the questionnaire. Participants retained the autonomy to withdraw from the process at any time, both before sending the form and after sending it.

### 2.2. Questionnaire

The link for the Questionnaire S1 was widely promoted. It was shared through the internal media of the Bauru School of Dentistry of the University of São Paulo as well as with all the research groups on popular social networks like Facebook (Version 482.1.0) and Instagram (Version 350.0.0). The questions on the form are listed below.

1. Gender—( ) Female ( ) Male ( ) Prefer Not To Answer.
2. Age—options with age groups.
3. Education—options with schooling.
4. Which region of Brazil do you live in?—options with Brazilian regions.
5. Have you taken any medication for pain and/or inflammation in the last 90 days?—options with the most common generic and trade names of anti-inflammatory/analgesic drugs.
6. For how many days?—day period options.
7. What was the main reason you took the medication?—options with the most common types of pain/inflammation.
8. Have you had any adverse reactions to the anti-inflammatory/analgesic?—options with the most common adverse reactions.
9. Have you taken any medication to treat an infection in the last 90 days?—options with the most common generic and trade names of antibiotics.
10. For how many days?—day interval options.
11. What was the main reason you took the medicine?—options with the most common types of infections.
12. Has this medicine been prescribed (this time) by a professional (doctor or dentist)? ( ) Yes ( ) No.
13. Have you had any adverse reactions to the antibiotic?—options with the most common adverse reactions.
14. Is there anything else you’d like to say?

Given that the questionnaire is a single one on the Google Forms platform, only those who answered all the questions were eligible to take part in the survey, since the configurations selected from the tools available on the platform were calibrated according to this requirement for this study.

## 3. Results

### 3.1. Sociodemographic Data

A total of 400 surveys were completed, with the majority of responses being submitted by female participants (71.8%). The predominant age group falls between 18 and 28 years (52.3%). Additionally, there is a varied range of educational attainment, with incomplete higher education being the most common (35%) followed by Postgraduate, Master’s, and PhD (26%), and Complete Higher Education (21.3%). The groups considered to have less education had low numbers of volunteers, such as Incomplete Elementary School (0.5%) and Complete Elementary School (0.5%). Concerning the distribution of data regarding the region of Brazil, the vast majority of volunteers were from the Southeast (88%), followed by the Central-West (8.8%) and Northeast (5.2%) regions. The demographic details of the participating volunteers are presented in the table below (Table 1).

### 3.2. Consumption of NSAIDs and Antibiotics by Volunteers

**Figure 1:**
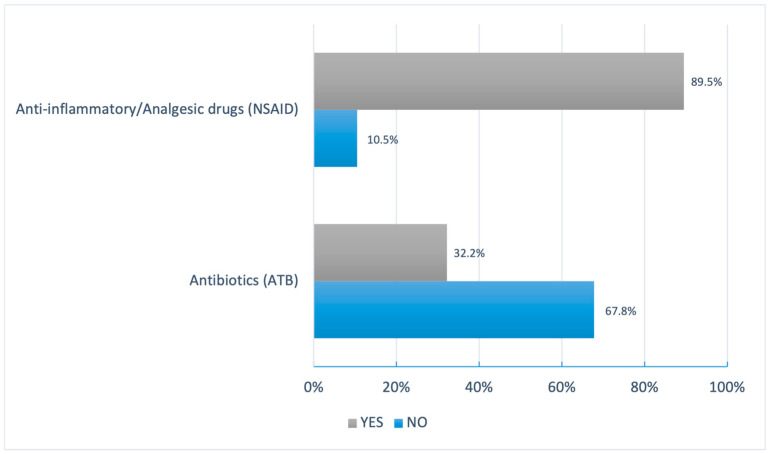
Relation of responses from volunteers who had or had not used anti-inflammatory/analgesic drugs and antibiotics in the last 90 days before the questionnaire (prescribed or not by medical doctors or dental surgeons).

In regard to the use of anti-inflammatory/analgesic drugs and antibiotics in the 90 days prior to answering the questionnaire, regardless of whether or not these drugs had been prescribed by doctors or dental surgeons, the vast majority of volunteers made use of anti-inflammatory/analgesic drugs (89.5%). Regarding antibiotics, the number of volunteers who said they had used this class of medication was lower than that of the aforementioned class (32.2%). Of these, approximately one-third of the participants who took antibiotics did so without a medical or dental prescription (8.8%). In addition, consumption for an inadequate period of time was also observed among the volunteers who used these drugs (1.5%). The following graph shows the groups of medicines analyzed in the survey, as well as their respective positive and negative percentages of use by the volunteer population (Figure 1).

**Figure 2:**
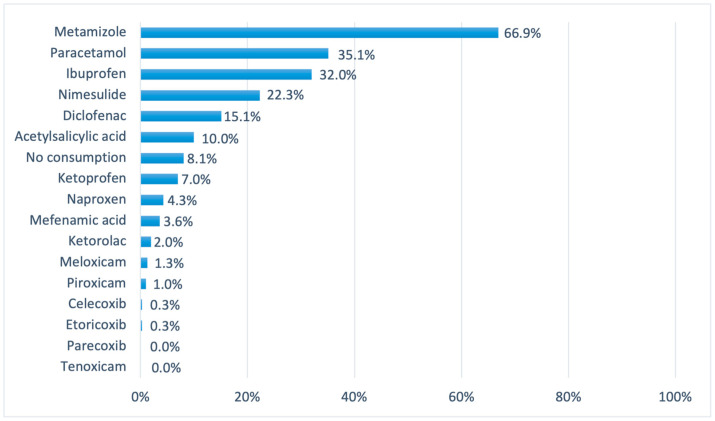
List of NSAIDs consumed by the volunteers, with independent percentages between groups.

In terms of drugs consumed, the volunteers had the opportunity to select one or more drugs that they had consumed in the 90 days preceding their answers to the questionnaire. Thus, the data for each drug generated in the graphs show an independent percentage for each drug selected, and there may be more than one chosen by the volunteers. That said, among the anti-inflammatory and analgesic medications mentioned, metamizole was the one most consumed by the participants (66.9%), followed by paracetamol (35.1%) and ibuprofen (32%). The data for each drug are presented graphically below (Figure 2).

As observed, the consumption of NSAIDs was considered very high, especially when related to the main reasons the volunteers used these medications, i.e., headaches (62%), muscle pain (36.5%), and colds or flu (27.3%).

**Figure 3:**
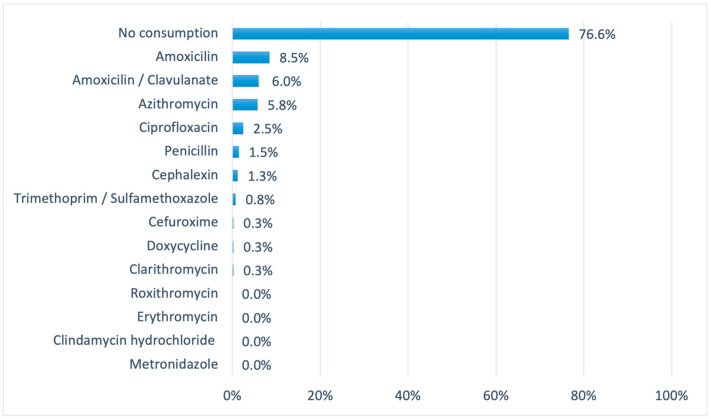
List of antibiotics consumed by the volunteers, with independent percentages between groups.

Regarding the consumption of antibiotics by participants, approximately 32.2% of volunteers reported using antibiotics in the last 90 days prior to the survey. Notably, the consumption of this class of drugs was not as high as the aforementioned, with amoxicillin being the most consumed (8.5%), followed by amoxicillin/clavulanate (6.0%) and azithromycin (5.8%). The complete data for each drug, as well as percentages, are shown graphically (Figure 3).

In addition, concerning the main reason why volunteers responded positively to taking antibiotics, the majority reported respiratory tract infections (7.8%), followed by genitourinary infections (3.3%). In addition, of all the volunteers who responded positively to the use of these medicines (32.2%), around one-third (8.8%) responded negatively to the prescription of the medicine by a professional. Moreover, it was noted that the length of time (in days) that the medication was taken varied considerably (0–2 days—1.5%; 3–5 days—7.3%; 7–9 days—12.5%; more than 10 days—2.7%).

### 3.3. Adverse Effects

**Figure 4:**
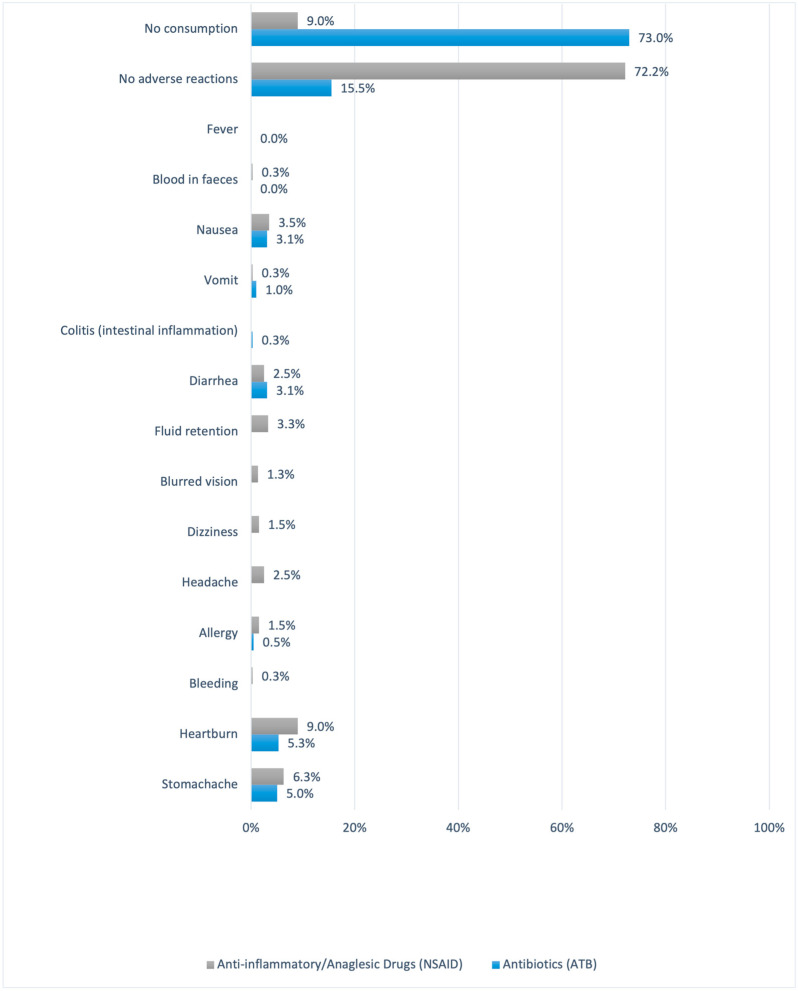
Adverse effects related to the consumption of NSAIDs and antibiotics.

Regarding the associated adverse effects, among the volunteers who responded positively about taking the drugs, around 19% had adverse effects in relation to anti-inflammatories and analgesics, and in relation to antibiotics, the number was near 12% of the sample. Among them, stomachache (6.3%—NSAIDs; 5%—ATB), heartburn (9%—NSAIDs; 5.3%—ATB), diarrhea (2.5%—NSAIDs; 3.1%—ATB) and nausea (3.5%—NSAIDs; 3.1%—ATB) were the most common for both anti-inflammatories and analgesics and antibiotics (Figure 4).

## 4. Discussion

The present study analyzed the behavior of volunteers regarding the use of NSAIDs and antibiotics in the Brazilian adult population during the period from June 2022 to June 2023. The socio-demographic data revealed a prevalence of young people with a high level of education. Other studies [26,27] have shown that, contrary to what one might think, young people with a higher level of education are more susceptible to self-medication. This could be associated with the fact that students have a greater ability to search for information about medicines on the internet and, as a result, to use these medications [26]. In addition, a large proportion of students self-prescribe medicines based on previous professional prescriptions according to previous individual experiences [27]. Several conducted studies have shown that in Brazil, the percentage of self-medication among students is higher than among adults who do not study, and this percentage is even higher when comparing the ratio between students from other areas and those from healthy areas [26,27].

Furthermore, the gender factor was taken into account due to the disparity between men and women as study volunteers. Studies have shown that, culturally, women tend to be more concerned about their health and general well-being than men. Women are often more socialized to attend more to their physical and emotional needs—a fact that has repercussions on health care, from visits to the doctor for preventive examinations and self-care to healthy behaviors and adherence to treatments for chronic conditions [28,29,30]. However, it is not conclusive that this figure reflects the entire population’s use of NSAIDs and antibiotics. Moreover, the study data revealed that the majority of volunteers who took part in the study were from the Southeast region of Brazil, because the Southeast and South are the most populous regions in the country and, accordingly, have the most access to information media. Thus, even with the wide dissemination of the questionnaire in all media, the Southeast region had the highest index in relation to the other regions, where the range may have been impaired.

The consumption of NSAIDs by the volunteers taking part in the survey was considered extremely high, given that the participants’ main reasons for using the drug were complaints related to headaches or muscle pain and colds or flu. Many studies have already shown the effectiveness of these drugs in managing pain and inflammation [6,31,32]. However, the misuse of NSAIDs as well as their chronic use is associated with various adverse effects, especially those related to the gastrointestinal tract [33]. As a result, this behavior becomes not only a problem for the individual but also for the health system in general. This is because NSAIDs are over-the-counter (OTC) medicines in Brazil—in other words, they do not require a medical or dental prescription to be bought in pharmacies. In addition, studies have already introduced the concept of a drug-related problem, which is defined as an event or circumstance involving drug therapy that can actually or potentially interfere with desired health outcomes. Consumers often consider OTC medicines to be harmless and use them for the time and in the quantities they believe to be effective, underestimating or unaware of the potential risks of the drug [34].

Regarding the consumption of antibiotics, this study showed that one-third of the percentage of volunteers who stated they used these medications did so without a doctor or dentist’s prescription. The main reason for taking these drugs was genitourinary and respiratory tract infections, probably related to the COVID-19 pandemic [35]. This could be a problem in terms of the misuse of this medication, through either over-medication or under-medication, as it is known that in Brazil, antibiotics are only sold with a prescription from a doctor or dentist, and nevertheless, some of the volunteers used these drugs without any kind of prescription. Antibiotic resistance refers to the ability of bacteria to survive the presence of the antibiotic intended to prevent or kill them [36,37]. The present study also revealed that the length of time (in days) that antibiotics were taken varied—from volunteers who took them for less than 3 days to volunteers who took them for more than 10 days. Other studies have already revealed that antibiotic resistance by bacteria is becoming a trend since the change in strategy of many treatments has decreased the effectiveness of these drugs in general. To remedy this problem, an approach has been developed to optimize the use of these drugs with an appropriate selection, dosage and duration time for each antimicrobial treatment. The program was designed to improve patient outcomes while reducing adverse effects and limiting the spread of resistant bacteria. One component that the researchers considered fundamental was the education and training of healthcare professionals in the principles of responsible antibiotic use. This included encouraging them to prescribe antibiotics only when necessary and to select the most appropriate agents based on local resistance patterns and individual patient needs [38].

Unfortunately, a limitation of this study was the disproportionate number of responses from the South and Southeast regions of Brazil. Nevertheless, efforts were made to engage volunteers from other regions of the country, recognizing its vast geographical and cultural diversity. Furthermore, the study also has another potential limitation in the method of distributing the questionnaire for participation in the survey, since when comparing the demographic data related to the age distribution of the volunteers, it can be seen that the percentage drops progressively as the age group increases.

The escalating trend of self-medication presents significant concerns for public health, necessitating the development of well-defined health policies. Engaging in self-medication without professional oversight can result in various adverse outcomes, including medication-related complications, hazardous drug interactions, and the exacerbation of existing health conditions. Addressing this issue requires the implementation of evidence-based health policies that account for societal, economic, and cultural influences impacting individuals’ medication practices. Educational initiatives must be deployed to raise public awareness regarding the risks associated with self-medication and to encourage the pursuit of appropriate medical guidance. Furthermore, stringent regulations governing the sale of non-prescription medications are imperative to ensure their responsible and safe distribution. Collaborative efforts involving healthcare professionals, pharmacists, and regulatory authorities are essential for the continuous monitoring and assessment of community medication utilization, facilitating timely interventions as necessary.

The widespread and excessive consumption of NSAIDs and antibiotics among the Brazilian population is a pressing concern. This behavior poses a significant threat not only to the individuals themselves but also to the overall integrity of the healthcare system and global public health. The improper and prolonged use of these medications not only leads to a range of adverse reactions but also contributes to the development of bacterial resistance, particularly in the case of antibiotics. This resistance can have profound implications for the effectiveness of antibiotic treatments and the ability to manage infectious diseases effectively.

When it comes to NSAIDs, it is essential to be aware of the possible risks associated with OTC medicines. While these drugs can provide relief for common ailments, they also carry the risk of adverse effects if not used properly. It is important that people understand the importance of reading and following the instructions on the labels of OTC medicines. In addition, it is essential to seek the advice of a healthcare professional before using these medicines, especially when it comes to chronic illnesses. Informing yourself about the possible risks of using over-the-counter medicines responsibly can help ensure the safety and well-being of each individual.

Despite the Ministry of Health’s efforts to regulate and implement norms and protocols for prescribing and using medicines in Brazil, unfortunately, adherence to these protocols is still considered very low in terms of the population’s proper consumption of medication, as we have observed in the results obtained in this study. The training of health professionals who have access to the distribution of these medicines must be reinforced so that this problem does not take on even more worrying proportions, thus resulting in a health management problem in the country.

In the current rapidly evolving digital landscape, it is necessary to assist the public in discerning the information they encounter. It is essential for individuals to be well versed in the intricacies of diseases, medications and treatments, and this knowledge must be accompanied by an astute awareness of the sought-after information. For example, in the context of self-medication, individuals should be cognizant of the potential adverse effects of medications when not under the guidance of qualified professionals who possess comprehensive knowledge of each drug’s specific dosage and usage.

## 5. Conclusions

The high prevalence of consumption of NSAIDs by the population studied has an impact on the worsening of adverse reactions associated with these drugs, particularly in the gastrointestinal tract. Regarding antibiotics, the problem of consumption without a prescription from doctors or dental surgeons contributes not only to the deterioration of the health system but also to the worsening of a global problem regarding the increase in bacterial resistance due to the indiscriminate consumption of this class of drugs.

## Figures and Tables

**Table 1:** Sociodemographic characteristics of the participants.

		Percentage (%)
Gender	Male	28.2%
	Female	71.8%
Age	18 to 28 years old	52.3%
	29 to 39 years old	18.5%
	40 to 50 years old	13.5%
	51 to 61 years old	10.3%
	62 years old and over	5.5%
Education	Incomplete Elementary School	0.5%
	Complete Elementary School	0.5%
	Incomplete High School	0.8%
	Complete High School	16%
	Incomplete Higher Education	35%
	Complete Higher Education	21.3%
	Postgraduate, Master’s and PhD	26%
Brazil region	South Region	3.8%
	Southeast Region	81%
	Central-West Region	8.8%
	North Region	1.2%
	Northeast Region	5.2%

## Data Availability

The original contributions presented in the study are included in the article material, further inquiries can be directed to the corresponding authors.

## Supplementary Materials

The following supporting information can be downloaded at: https://www.mdpi.com/article/10.3390/pharmacy12050150/s1, Questionnaire S1: Anti-inflammatory and Antibiotic Form.
