# Supplementary material for: Study on the Consumption of Non-Steroidal Anti-Inflammatory Drugs and Antibiotics by the Brazilian Adult Population: A Cohort Study
Source: Pharmacy (Basel). 2024 Sep 29;12(5):150. doi: 10.3390/pharmacy12050150 (PMC11510564; doi:10.3390/pharmacy12050150)
Supplement: Supplementary file 1 [file pharmacy-12-00150-s001.zip › pharmacy-3183367-supplementary.pdf]

Anti-inflammatory and Antibiotic Form

RESEARCH PROJECT - ‘Study on the consumption of non-steroidal anti-inflammatory drugs (NSAIDs) and antibiotics by the Brazilian adult population’

Research project for scientific initiation in Pharmacology, carried out at the Bauru School of Dentistry, University of São Paulo

Dear research participant,
This is an invitation to take part in a research project being carried out by Fernanda Sagioneti and Sophia Iris Mendes do Nascimento, under the supervision of Prof Dr Adriana Calvo, undergraduate dental students at the Bauru Dental School, University of São Paulo.
The aim of the study is to find out about the consumption of antibiotics and anti-inflammatory/analgesics by the adult Brazilian population, taking into account the 90 days preceding the questionnaire, defining which medicines were most used, which were prescribed by a doctor or dentist, and which were consumed as self-medication, the period they were taken and the frequency of consumption.
Your participation comprises answering a form and the estimated time to complete it is approximately 10 minutes. Participation in this research is voluntary. Participants' personal data will be kept strictly confidential and used only for the purposes proposed in this study. All the necessary instructions are on the form.
Don't forget to click ‘SEND/SUBMIT’ when you have finished so that we can receive your response.
If you have any questions, please contact: fersagioneti@usp.br or sophiamendes@usp.br Thank you in advance for your cooperation.

* Indicates a mandatory question

E-mail *

____

TERM OF FREE AND INFORMED CONSENT

You are being invited as a volunteer to take part in the research entitled ‘Study on the consumption of non-steroidal anti-inflammatory drugs (NSAIDs) and antibiotics by the Brazilian adult population’, authored by Professor Adriana Maria Calvo and undergraduate students Fernanda Teixeira Sagioneti and Sophia Iris Mendes do Nascimento, from the Bauru School of Dentistry - University of São Paulo (FOB/USP).

After reading the form, you will be able to ask questions about each stage and decide whether or not you would like to take part in the research described.
The aim of this survey is to find out about the pattern of consumption of medicines popularly used by the Brazilian population, such as antibiotics and anti-inflammatory/analgesics in the last 90 days prior to filling in the form. In addition, through the survey that will be obtained, the importance of public policies on knowledge of medicines and the importance of correct prescriptions for each case will be reinforced.

If you agree to take part in this research, your cooperation will consist of filling in a simple form about the medicines you have taken in the last three (3) months, whether on medical or dental advice or as self-medication.

The participants will not benefit directly from the results of this research. However, with the conclusion of this research, we will be able to indirectly benefit the Brazilian population with public policies to raise awareness of the risks of self-medication and the correct prescription of these medicines for the appropriate uses.

The hassle related to this survey consists of filling in 15 questions that will take approximately 10 minutes to answer.

The risks of identifying the answers are minimal since, in addition to the confidentiality of the data filled in and coded by the researchers, this data will be sent via secondary sources using the form.

It is important to emphasize that you will not be paid and will not incur any costs for taking part in the research and that you have legal guarantees of compensation if you are harmed in any way.

You are also free to refuse to take part in the research or to discontinue your participation at any time, for any reason, without any prejudice or loss of benefits. Your personal data will be kept strictly confidential and will only be used in this study.

You can ask any questions you may have by contacting us below.
Names, addresses, and telephone numbers of those responsible for the study, to contact if you have any questions: Prof Adriana (14) 99111-0074, Fernanda (17) 99192-1625, fersagioneti@usp.br and Sophia (14) 99889-1605, sophiamendes@usp.br, or Alameda Octávio Pinheiro Brisolla 9-75, CEP 17012-901, Disciplina de Farmacologia, Faculdade de Odontologia de Bauru/USP. If you have any complaints, please contact the coordinator of the Research Ethics Committee of the Bauru School of Dentistry (FOB/USP), at Alameda Octávio Pinheiro Brisolla 9-75, Vila Universitária, Bauru, or call (14) 3235-8356, e-mail: cep@fob.usp.br.
Finally, as the researcher responsible for the study, I DECLARE compliance with the provisions of CNA Resolution no. 466 of 2012, contained in items IV.3 and IV.5.a and in full with CNS Resolution no. 466 of 12 December 2012.

The Research Ethics Committee - CEP, organized and created by FOB-USP on 29/06/98 (Ordinance GD/0698/FOB), as provided for in item VII of CNS Resolution 466/12 of the National Health Council of the Ministry of Health (published in the DOU of 13/06/2013), is an interdisciplinary and independent, publicly relevant, advisory, deliberative and educational body, created to defend the interests of research participants in their integrity and dignity and to contribute to the development of research within ethical standards.
Any complaints about your participation in the research can be reported to this CEP:

Opening hours and location:
Research Ethics Committee
Bauru School of Dentistry USP - Postgraduate Building (block E - upper floor), Monday to Friday (on working days), from 14hs to 17h30.
Alameda Dr Octávio Pinheiro Brisolla, 9-75
Vila Universitária - Bauru - SP - CEP 17012-901
Phone/FAX(14)3235-8356
e-mail: cep@fob.usp.br

After thoroughly reading the information contained in this FREE AND INFORMED CONSENT FORM, duly explained in the text in its smallest detail, aware of the procedures to which you will be subjected, leaving no doubts about what you have read and explained, please DECLARE and FIRM your FREE AND INFORMED CONSENT by agreeing to participate in the proposed research voluntarily.
It is understood that the research participant can withdraw their FREE AND INFORMED CONSENT at any time and stop taking part in this research, in the knowledge that all information provided will be kept confidential by virtue of professional secrecy (Chap. III, Art. 9 of the Code of Dental Ethics (Res. CFO-118/2012).

*

Mark only one oval.

- ⬭ I have read and agree to take part in the study
- ⬭ I don't agree to take part in the study

Full Name *

____

Document number (RG) *

____

Your email: *

____

Questions

Multiple choice tests

1) Gender *

Mark only one oval.

- Female
- Male
- Other: ______________________________

2) Age *

Mark only one oval.

- 18 years to 28 years
- 29 years to 39 years
- 40 years to 50 years
- 51 years to 61 years
- Over 62

3) Education *

Mark only one oval.

- Incomplete Elementary School
- Complete Elementary School
- Incomplete High School
- Complete High School
- Incomplete Higher Education
- Complete Higher Education
- Postgraduate, Master’s and PhD

4) Which region of Brazil do you live in? *

Mark only one oval.

- Southern Region
- Southeast Region
- Central-West Region
- Northern Region
- Northeast Region

5) Have you taken any medication for pain and/or inflammation (anti-inflammatory/analgesic) in the last 90 days?

Mark only one oval.

- Yes
- No

6) Which of the following drugs have you taken for pain and/or inflammation? *

Check all that apply.

- [ ] No consumption
- [ ] Aspirin, ASA, Engov, Sonrisal
- [ ] Paracetamol, Tylenol, Sonridor, Termol
- [ ] Dipyrone, Neosaldine, Magnopyrol, Benegrip, Buscopan, Dorflex, Anador, Atroveran
- [ ] Ibuprofen, Advil, Lombalgina, Ibupril, Ibuprofan
- [ ] Ceprofen, Cetoprofen, Flamador, Profenid
- [ ] Naproxen, Flanax, Naprox
- [ ] Diclofenac, Cataflam, Torsilax, Voltaren
- [ ] Ketorolac, Toragesic
- [ ] Piroxicam, Feldene, Floxicam, Piroxin
- [ ] Tenoxicam, Tilatil, Tilonax
- [ ] Meloxicam, Artitec, Bioflac, Movatec
- [ ] Nimesulide, Nisulid, Maxsulid
- [ ] Celebra
- [ ] Arcoxia
- [ ] Bextra
- [ ] Mefenamic acid

7) For how many days ? *

Mark only one oval.

- ( ) No consumption
- ( ) 0-2 days
- ( ) 3-5 days
- ( ) 7-9 days
- ( ) more than10

8) What was the main reason you took the anti-inflammatory/analgesic? *

Check all that apply.

- [ ] No consumption
- [ ] Dental pain
- [ ] Muscle pain
- [ ] Headache
- [ ] Flu/cold
- [ ] Fever
- [ ] COVID
- [ ] Articular pain
- [ ] Menstrual cramps
- [ ] Renal colic
- [ ] Postoperative surgery

Other: ______________________

9) Have you had any adverse reactions to the anti-inflammatory/analgesic? *

Check all that apply.

- [ ] Stomachache
- [ ] Heartburn
- [ ] Gastritis
- [ ] Bleeding
- [ ] Allergy
- [ ] Headache
- [ ] Dizziness
- [ ] Blurred vision
- [ ] Fluid retention
- [ ] Diarrhea
- [ ] Vomit
- [ ] Nausea
- [ ] Blood in feces
- [ ] No adverse reactions
- [ ] No consumption
- [ ] Other: ______________________

10) Have you taken any medication to treat infection (antibiotics) in the last 90 days?

Mark only one oval.

- Yes
- No

11) Which of the following medicines have you taken to treat infection in the last 90 days?

Mark all that apply.

- [ ] No consumption
- [ ] Benzetacil, Pen-Ve-Oral, Penicilin
- [ ] Amoxil, Amoxicillin, Ampicil
- [ ] Amoxicillin with Clavulanate, Clavulin, Clavamox
- [ ] Cephalexin, Maxcef
- [ ] Azithromycin, Zithromax, Azi
- [ ] Clarithromycin, Klaricid
- [ ] Roxithromycin, Rulid
- [ ] Erythromycin, Ilosone
- [ ] Doxycycline, Vibramycin, Tetracycline
- [ ] Dalacin C
- [ ] Ciprofloxacin, Cipro, Levofloxacin
- [ ] Bactrim, Bactrim F
- [ ] Flagyl
- [ ] Cefuroxime, Zinnat

Other: ____________________

12) For how many days ? *

Mark only one oval.

- No consumption
- 0-2 days
- 3-5 days
- 7-9 days
- more than 10

13) What was the main reason you took the antibiotic ? *

Mark all that apply.

- [ ] No consumption
- [ ] Dental infecctions
- [ ] Gastric infecctions
- [ ] Urinary tract infecctions
- [ ] Respiratory infecctions
- [ ] Skin infecctions
- [ ] COVID
- [ ] Postoperative surgery

Other: ____________________

14) Has this medicine been prescribed (this time) by a professional (medcal doctor or dentist)?

Mark only one oval.

- Yes
- No
- No consumption

5) Have you had any adverse reactions to the antibiotic? *

Mark all that apply.

- [ ] Stomachache
- [ ] Heartburn
- [ ] Gastritis
- [ ] Diarrhea
- [ ] Allergy
- [ ] Colitis (intestinal inflammation)
- [ ] Nausea
- [ ] Vomit
- [ ] Blood in feces
- [ ] Fever
- [ ] No adverse reactions
- [ ] No consumptiom
- [ ] Other: ________________

Do you have any other comments?

.

Google Forms
